# Development and characterization of genomic SSR markers for *Tamarix chinensis* (Tamaricaceae)

**DOI:** 10.1002/aps3.1219

**Published:** 2019-01-30

**Authors:** Ruhua Zhang, Qiang Wen, Li‐an Xu

**Affiliations:** ^1^ College of Life Sciences Linyi University Linyi Shandong 276005 People's Republic of China; ^2^ Jianxi Academy of Forestry Nanchang Jiangxi 330032 People's Republic of China; ^3^ Key Laboratory of Forest Genetics and Biotechnology Nanjing Forestry University Ministry of Education Nanjing 210037 People's Republic of China

**Keywords:** cross‐species amplification, Illumina sequencing, simple sequence repeat (SSR) markers, Tamaricaceae, *Tamarix chinensis*

## Abstract

**Premise of the Study:**

We developed a set of microsatellite markers to study the population genetic structure, mating system, and interspecific hybridization of *Tamarix chinensis* (Tamaricaceae), an alkali‐ and salt‐tolerant shrub endemic to China, Korea, and Japan.

**Methods and Results:**

Using Illumina sequencing, we developed 10 polymorphic and 11 monomorphic microsatellite primers. High levels of polymorphism were detected in four *T. chinensis* populations. Allele numbers ranged from two to 11, and the levels of observed and expected heterozygosity ranged from 0.182 to 0.846 and from 0.165 to 0.794, respectively. The polymorphism information content values ranged from 0.201 to 0.803. Cross‐species amplification showed two to 15 alleles per locus in 24 individuals from one natural population of the congener *T. ramosissima*, and the levels of observed and expected heterozygosity ranged from 0.042 to 0.864 and from 0.041 to 0.892, respectively.

**Conclusions:**

These markers should be useful for exploring the population genetic structure, mating system, and gene flow of *T. chinensis*.

Belonging to the family Tamaricaceae, *Tamarix chinensis* Lour. is an alkali‐ and salt‐tolerant, loosely branched deciduous shrub or 3–6‐m‐tall tree. It is naturally distributed from the temperate to subtropical zones in China, Korea, and Japan, inhabiting riverbeds, sandy floodplains, deserts, and coastal tidal flats (Baum, 1978) and has been naturalized in much of the western United States since its introduction in the mid‐nineteenth century (Whitcraft et al., 2007). The level of genetic diversity and mating system of the species are not well known. A few studies have focused on the genetic diversity and population genetic structure of *T. chinensis* using random amplified polymorphic DNA (RAPD) (Zhao et al., 2008) and inter‐simple sequence repeat (ISSR) markers (Jiang et al., 2011). Codominant simple sequence repeat (SSR) markers are powerful tools for population variation analysis and for the estimation of gene flow through genotypic exclusion in a number of tree species (Vahdati et al., 2015). To date, expressed sequence tag (EST)–SSR markers have been developed through mining of EST databases of *Tamarix* L. spp., and the transferability across species including *T. chinensis*,* T. gallica* L., *T. aphylla* (L.) H. Karst, *T. jordanis* Boiss., *T. nilotica* (Ehrenb.) Bunge, and *T. tetragyna* Ehrenb. was tested (Terzoli et al., 2013). A set of 10 genomic microsatellites of *T. ramosissima* Ledeb. was isolated using the biotinylated‐oligonucleotide capture method (Gaskin et al., 2006), two of which showed polymorphism in Chinese *T. chinensis* material (Zhang, 2011). Although microsatellite primers have been developed for some *Tamarix* spp., none have yet been identified specifically for *T. chinensis*. We obtained a large number of DNA sequences based on high‐throughput sequencing and characterized SSRs distributed in the genome of *T. chinensis*. A set of 10 polymorphic SSR molecular markers of *T. chinensis* were developed and the transferability was tested in its congener *T. ramosissima*.

## METHODS AND RESULTS

Fresh leaves of one *T. chinensis* individual from a natural population of Binzhou City, Shandong Province, China (Appendix 1), were collected for DNA extraction and Illumina sequencing. Genomic DNA was extracted using a DNeasy kit (QIAGEN, Venlo, The Netherlands). Genomic libraries were constructed using the method for RAD sequencing as described by Baird et al. (2008). A Qubit 2.0 kit (Thermo Fisher Scientific, Waltham, Massachusetts, USA) was used to evaluate the quality of the libraries, and the Agilent 2100 bioanalyzer (Agilent Technologies, Santa Clara, California, USA) was used to check the sizes of the libraries after they were diluted to 1 ng/μL. Sequencing was conducted on the HiSeq 2500 high‐throughput sequencing system (Illumina, San Diego, California, USA) to generate 125‐bp paired‐end reads by a commercial company (Novogene Co. Ltd., Beijing, China). Raw data were cleaned up by trimming the adapters and low‐quality reads with a custom script by the company who did the sequencing, and by filtering reads with read depth of <10 and >400 using CD‐HIT‐EST (Li and Godzik, 2006) to avoid false positives.

Assembly of paired reads was performed using Velvet version 1.1.06 (Zerbino and Birney, 2008). Contigs with a length of less than 125 bp were deleted. Raw sequences were deposited to the National Center for Biotechnology Information (NCBI) Sequence Read Archive (SRA accession PRJNA492209). A total of 818,142 contigs were generated with an average length of 323 bp and an N50 length of 474 bp. The MISA perl script (Thiel et al., 2003) was used to search for SSRs with mono‐, di‐, tri‐, tetra‐, penta‐, and hexanucleotide motifs with a minimum repeat number of 14, eight, six, five, four, and four, respectively. Compound microsatellites were defined as having two or more motifs separated by an interval of ≤100 bp. A total of 31,140 SSRs were identified using the MISA perl script for 28,454 contigs. There were 2567 contigs that contained more than one SSR, and there were 2027 compound SSRs. Di‐ and trinucleotide motifs were the most abundant, comprising 41.53% and 41.24%, respectively. SSR primers were designed with Primer3 version 1.1.4 (Rozen and Skaletsky, 1999) with the following qualifications: primer length range from 18 to 22 bp and annealing temperature 55–60°C. Twenty‐four primers were synthesized by a commercial company (GenScript, Nanjing, China).

In total, 58 individuals of *T. chinensis* from four natural populations and 24 individuals of *T. ramosissima* from one population were sampled (Appendix 1). We collected no more than 20 individuals for each *T. chinensis* population because the sampled *T. chinensis* individuals were scattered along riverbanks, often 50 m apart, and some individuals were damaged. Genomic DNA was extracted with the DNeasy kit (QIAGEN) and tested on agarose gel.

As an initial primer test, two individuals were randomly chosen from each of the four studied *T. chinensis* populations (Appendix 1) to run PCR using the 24 primer pairs. The PCR had a 10‐μL volume and contained: 1 μL of 1× buffer (Tiangen, Beijing, China), 1 μL of MgCl_2_ (20 mM), 1 μL of dNTPs (2.5 mM each), 0.2 μL of forward primer (10 μM), 0.2 μL of reverse primer (10 μM), 0.2 μL of *Taq* polymerase (5 U/μL) (TaKaRa Bio Inc., Kyoto, Japan), 0.5 μL of DNA (50 ng/μL), and 5.9 μL of ddH_2_O. The PCR profile was as follows: 4 min denaturation at 94°C; followed by 30 cycles of 30 s denaturation at 94°C, 30 s annealing step at 55°C, and 30 s elongation at 72°C; and a final extension step at 72°C for 3 min. PCR products were tested by polyacrylamide gel electrophoresis (PAGE) as an initial polymorphism test. The verified polymorphic primers (altogether 10 polymorphic SSRs, Table 1) were synthesized by GenScript (Nanjing, China) with forward primers labeled with 6‐FAM. The fluorescent‐labeled primers were used to run PCR in 58 *T. chinensis* individuals and 24 *T. ramosissima* individuals using the reaction volume and PCR profile as described above. PCR products combined with deionized formamide, using GeneScan 500 LIZ (Thermo Fisher Scientific) as a size standard, were run on a 3730 Genetic Analyzer (Thermo Fisher Scientific), and alleles were assigned to bins in GeneMarker version 2.6.7 (SoftGenetics, State College, Pennsylvania, USA). The levels of observed (*H*_o_) and expected heterozygosity (*H*_e_) and number of alleles were calculated in GenAlEx 6.502 (Peakall and Smouse, 2005). The GENEPOP allele .txt file was exported from GenAlEx for Hardy–Weinberg equilibrium (HWE) and linkage disequilibrium analysis in GENEPOP (version 1.2) (Raymond and Rousset, 1995). The polymorphism information content (PIC) value of each locus was calculated in CERVUS version 3.0.3 (Kalinowski et al., 2007), according to the following formula: $PIC=1-\sum_{i=1}^{n}f_{i}^{2}$, in which *f*_*i*_ is the frequency of the *i*th allele and *n* is the allele number. Significance levels were tested with sequential Bonferroni corrections (Rice, 1989).

**Table 1:** Characteristics of 21 novel genomic microsatellite markers developed for *Tamarix chinensis*

Locus^a^	Primer sequences (5′–3′)	Repeat motif	Allele size range (bp)		GenBank accession no.
			*T. chinensis*	*T. ramosissima*	
TC1	F: ATGTGGGGAGGTGGAGTG	(CTT)_10_	115–127	115–121	MG856343
	R: AATGTATGCAGACAAAAGT				
TC3	F: AAAGCAGGTGAGATTGAA	(TTA)_11_	150–204	153–201	MG856344
	R: ACACCCTAATCCACATAAC				
TC4	F: TATCCCGAGGTTGTAAAT	(AAT)_11_	162–201	162–186	MG856345
	R: GCTGCTGGTCACCACTAA				
TC5	F: GTCTGCCTAAGAAGTCGC	(TCTT)_8_	189–221	186–218	MG856346
	R: CGGAAATAAGGGAGAAAT				
TC6	F: GATAAGGTTTTGACGATT	(ATA)_11_	158–236	158–230	MG856347
	R: TCTAGTCACCACCATCCC				
TC7	F: GGTCCTTTAGGTTCTTCC	(TATT)_8_	194–214	194–206	MG856348
	R: TATGGCCTCAACTATCTT				
TC8	F: TTTGAGTTTGACGATGTA	(AAT)_11_	208–244	208–238	MG856349
	R: GATTGACCGTGTTTTAGT				
TC12	F: TAAGAAGGGTAGAGGAGA	(AAG)_11_	281–341	281–338	MG856350
	R: TAATCAATAGTCACAAGG				
TC17	F: AGTAGAGGCAAAGGTTAT	(TGA)_10_	335–347	332–344	MG856351
	R: CTCAAAAGTCCCTCATAG				
TC19	F: GAGGGTGGGCAAGAAATG	(TTTA)_8_	369–409	369–409	MG856352
	R: TGACGCAGCAGTAGTGTA				
TC2	F: CATTGTCATCATCCCACT	(AAT)_10_	151	151	MG856353
	R: TCTTGTTGCCGACTTTGT				
TC9	F: CGAAACTAATAACCCTAA	(TAA)_10_	205	205	MG856354
	R: CTATCCCTGCCGACTCAA				
TC10	F: CAACTTTCCACCCTTCTT	(ATT)_11_	239	239	MG856355
	R: ATTCCGAGGCTACACTTA				
TC11	F: CAGTGTTATTGAAGGGTT	(TTA)_10_	256	256	MG856356
	R: GATTGTTGATGCGGATGG				
TC13	F: TTCTAACCCAAAACACTC	(TATT)_8_	273	273	MG856357
	R: ATGAAAATCCTTCCTTGT				
TC14	F: AAATGATGTGCTTGTCGT	(AAG)_10_	290	290	MG856358
	R: TTTAATAGCTTCTTGGAG				
TC15	F: CTTAGCCTTAGCACTTGG	(TAT)_11_	317	317	MG856359
	R: TAACTTCCCTCTTACTCC				
TC16	F: TCTTCGGGTTGAGATTAC	(CATA)_10_	320	320	MG856360
	R: TAAGGGCTTGTTTGGGAG				
TC21	F: ATAATCTCCACCCTGCCAACA	(ATT)_11_	382	382	MG856361
	R: AACCACCATCCACTACCACATC				
TC22	F: TCCTCTACCCTTTCTTGC	(AAT)_10_	383	383	MG856362
	R: ATTCCCAGTTCCACCACA				
TC24	F: TTAATGCAGTCACGAGTT	(ATT)_10_	409	409	MG856363
	R: GTGGTAATGTGGACGAAT				

a Annealing temperature for all loci was 55°C.

In total, 21 (GenBank accession no.: MG856343–MG856363) out of 24 primers amplified the target sequences. Of these, 11 were monomorphic and 10 were polymorphic in the PAGE test (Table 1). The 10 polymorphic primers showed high polymorphism in both species (Table 2). For *T. chinensis*, the allele number per locus per population varied from two to 11, with the highest average allele number (6.2) for the Kenli (KL) population, followed by Fangshan (FS) (5.6), Lijin (LJ) (5.2), and Helou (HK) (4.3). Levels of *H*_o_ and *H*_e_ per locus for each population ranged from 0.182 to 0.846 and 0.165 to 0.794, respectively, with the highest average *H*_e_ for the FS population (0.577), followed by KL (0.543), HK (0.542), and LJ (0.534). The PIC values for each locus ranged from 0.201 to 0.803.

**Table 2:** Level of polymorphism of 10 microsatellite loci developed for *Tamarix chinensis* in four *T. chinensis* populations and one *T. ramosissima* population.^a^

Locus	*T. chinensis*																*T. ramosissima*			
	KL (*N* = 18)				HK (*N* = 13)				LJ (*N* = 13)				FS (*N* = 14)				CY (*N* = 24)			
	*A*	*H*_o_	*H*_e_	PIC	*A*	*H*_o_	*H*_e_	PIC	*A*	*H*_o_	*H*_e_	PIC	*A*	*H*_o_	*H*_e_	PIC	*A*	*H*_o_	*H*_e_	PIC
TC1	3	0.558	0.517	0.469	3	0.364	0.310	0.478	3	0.556	0.526	0.594	3	0.300	0.455	0.566	3	0.387	0.457	0.399
TC3	10	0.812	o.794	0.803	5	0.677	0.593	0.612	7	0.524	0.616	0.673	8	0.626	0.723	0.717	13	0.864	0.892	0.882
TC4	4	0.414	0.507	0.516	3	0.337	0.415	0.468	4	0.384*	0.497	0.404	5	0.414*	0.535	0.487	6	0.591	0.685	0.610
TC5	6	0.667	0.583	0.632	4	0.660	0.678	0.669	7	0.750	0.726	0.723	4	0.556	0.562	0.605	8	0.739	0.812	0.788
TC6	11	0.607	0.733	0.645	7	0.541	0.501	0.627	9	0.653	0.582	0.605	11	0.549	0.645	0.653	15	0.609*	0.772	0.745
TC7	4	0.632	0.568	0.603	3	0.548	0.537	0.606	4	0.621	0.678	0.717	3	0.523	0.585	0.568	4	0.667	0.621	0.546
TC8	8	0.717	0.634	0.622	6	0.562	0.667	0.636	7	0.613	0.546	0.608	8	0.701	0.757	0.628	10	0.735	0.819	0.791
TC12	8	0.596	0.515	0.502	6	0.659	0.553	0.597	6	0.549	0.512	0.504	7	0.624	0.657	0.663	10	0.712	0.732	0.713
TC17	3	0.222	0.204	0.264	3	0.283*	0.434	0.310	2	0.182	0.165	0.201	3	0.231	0.210	0.385	2	0.042	0.041	0.040
TC19	5	0.706	0.630	0.643	3	0.846*	0.541	0.512	3	0.538	0.494	0.584	4	0.636	0.645	0.693	8	0.708	0.667	0.612
Mean	6.2	0.593	0.543	0.570	4.3	0.548	0.542	0.572	5.2	0.537	0.534	0.561	5.6	0.516	0.577	0.597	7.9	0.605	0.650	0.601

Note

*A* = number of alleles; *H*_e_ = expected heterozygosity; *H*_o_ = observed heterozygosity; *N* = sample size for each population; PIC = polymorphism information content.

a Voucher and locality information are provided in Appendix 1.

*Indicates that *H*_o_ departs significantly from *H*_e_ under Hardy–Weinberg equilibrium (*P* < 0.01).

For *T. ramosissima*, the allele number per locus varied from two to 15, with an average allele number per locus of 7.9. Levels of *H*_o_ and *H*_e_ ranged from 0.042 to 0.864 and 0.041 to 0.892, with an average *H*_o_ and *H*_e_ of 0.605 and 0.650, respectively. The PIC values ranged from 0.040 to 0.882. HWE testing revealed no loci (for KL), one locus (for LJ and FS), and two loci (for HK) for *T. chinensis*; for the single population of *T. ramosissima* one locus deviated from equilibrium significantly (*P* < 0.01; Table 2). No significant linkage disequilibrium was detected between markers after Bonferroni correction for both species.

## CONCLUSIONS

This is the first report of genomic microsatellites for *T. chinensis*. The 10 polymorphic markers showed comparatively high genetic variation, transferability to congeneric species, little or no deviation from HWE, and were in linkage equilibrium. These properties make them especially useful for genetic analysis of population genetic structure, mating system, and gene flow in *T. chinensis* and its congeners.

## Data Availability

Raw sequences were deposited to the National Center for Biotechnology Information (NCBI) Sequence Read Archive (SRA accession PRJNA492209). Sequence information for the developed primers has been deposited to NCBI; GenBank accession numbers are provided in Table 1.

## APPENDIX 1 Voucher and locality information for *Tamarix chinensis* and *T. ramosissima* used in this study.^a^^,^ b

**Table:**

Species	Locality	Geographic coordinates	Population code	*N*	Voucher specimen accession no.^c^
*Tamarix chinensis* Lour.	Kenli, Shandong, China	37°48′06″N, 119°02′17″E	KL	18	Tch‐KL01‐ZR
*T. chinensis*	Hekou, Shandong, China	38°13′19″N, 118°50′30″E	HK	13	Tch‐HK02‐ZR
*T. chinensis*	Lijing, Shandong, China	38°02′13″N, 118°44′30″E	LJ	13	Tch‐LJ03‐ZR
*T. chinensis*	Fangshan, Beijing, China	39°63′19″N, 115°78′30″E	FS	14	Tch‐FS04‐ZR
*T. chinensis* d	Binzhou, Shandong, China	37°22′16″N, 118°03′22″E	BZ	1	Tch‐BZ05‐ZR
*T. ramosissima* Ledeb.	Changyi, Shandong, China	37°05′16″N, 119°21′22″E	CY	24	Tra‐CY01‐ZR

Note

*N* = number of individuals sampled.

a Vouchers are deposited in Linyi University, Shandong Province, China.

b All individuals were sampled from natural stands.

c ZR = Ruhua Zhang.

d Individual used for DNA extraction and Illumina sequencing.
